# Panic Disorder and Agoraphobia

**DOI:** 10.1192/pb.bp.114.047803

**Published:** 2015-04

**Authors:** Emma Barrow

**Figure:**
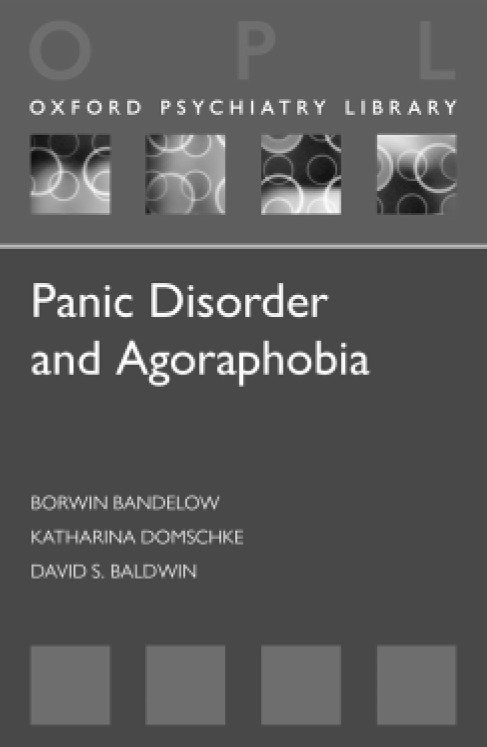


This text is part of the Oxford Psychiatry Library series and at about 80 pages there is nothing anxiety-provoking about its length. The authors make clear from the outset that this is a book designed to be user friendly in its approach and, with four independently referenced chapters, the reader instantly feels it will live up to this claim.

Published in the USA, the opening chapter on diagnosis refers to the (at the time of print) current DSM-IV and new DSM-5 diagnostic criteria. The main focus of diagnosis is, however, referencing the complex interplay between patients with panic disorder who frequently present with multiple and varied somatic complaints to a wide range of medical, surgical and psychiatric specialties. Of particular interest to this reader was the comprehensive table of medical conditions that can mimic panic attacks. Concise text is interspersed with useful boxes and tables, drawing the reader’s eye to the key points and considerations. Three further chapters on aetiology, pharmacological treatment and non-pharmacological treatment follow a similarly structured pattern.

For those who are looking for a summary of the current research into these disorders the ‘aetiology’ section on neurochemistry will not disappoint. For those, perhaps from non-psychiatry-based disciplines, looking to jump to more practical management advice, the section on ‘pharmacological treatment’ contains a handy FAQ list.

This book is by no means exhaustive, but what it quite cleverly manages to do is educate and interest the reader while guiding them through the practicalities of treating patients with panic disorder and agoraphobia in clinical practice. The section on pharmacological treatments is not as detailed as, say, the NICE or Maudsley guidelines, but its handy ‘pocket’ size means it is a worthy supplement and far more likely to be carried around. I have already found it useful, not only in my out-patient clinics but also in the acute care setting while on call – where it is important to bear in mind the reminder that appropriately placed psychoeducation can go a long way.

